# Circularly Polarized Ultra-Wideband Antenna for Uni-Traveling-Carrier Photodiode Terahertz Source

**DOI:** 10.3390/s23239398

**Published:** 2023-11-25

**Authors:** Qi Li, Chuang Nie, Zihao Liu, Xin Zhou, Xiaohe Cheng, Song Liang, Yuan Yao

**Affiliations:** 1 School of Electronic Engineering, Beijing University of Posts and Telecommunications, Beijing 100876, China; qi_li@bupt.edu.cn (Q.L.); niechuang@bupt.edu.cn (C.N.); liu.zihao@bupt.edu.cn (Z.L.); xinzhou@bupt.edu.cn (X.Z.); xiaohec@bupt.edu.cn (X.C.); 2 Key Laboratory of Semiconductor Materials Science, Institute of Semiconductors, Chinese Academy of Sciences, Beijing 100083, China; liangsong@semi.ac.cn

**Keywords:** circularly polarized antenna, UWB, UTC-PD, THz source

## Abstract

This paper proposes a circularly polarized ultra-wideband (UWB) antenna for a Uni-Traveling-Carrier Photodiode (UTC-PD) to meet the growing demand for bandwidth and polarization diversity in terahertz (THz) communication. In the design of the UTC-PD integrated antenna, the planar electrodes of the chip are directly integrated with the antenna to simplify the integration process. However, this integration introduces new problems, such as asymmetry inside the spiral antenna, which leads to a deterioration in the corresponding high-frequency performance. To address this issue, the antenna’s structure is optimized, and a lens is integrated to enhance directivity and eliminate surface waves. As a result, the proposed antenna achieves a 100–1500 GHz (175%) impedance bandwidth and a 150–720 GHz (131%) axial ratio bandwidth for the UTC-PD. The maximum gain of the antenna is 21.05 dBi at 1 THz.

## 1. Introduction

THz waves, which lie between millimeter waves and infrared light, possess distinctive characteristics that render them widely used in diverse applications such as security checks, imaging, and wireless communication [1,2,3]. The THz source is a crucial component of THz technology. Photodiode-based THz antennas also serve as significant THz sources due to their advantages of continuous operation at room temperature, broadband tuning, and high spectral purity. The PIN-PD (Positive–Intrinsic–Negative Photodiode) and UTC-PD are typical examples [4,5]. In PIN-PDs, both electrons and holes act as active carriers; however, the hole transport speed is considerably slower than that of electrons. Consequently, this leads to a decrease in response speed, poor high-frequency characteristics, and easy saturation under a certain bias [6,7].

In comparison, the UTC-PD has only electrons as active carriers, enabling rapid response and high saturation output power [8,9,10]. In a previous study [11], the integration of a UTC-PD with two types of slot antennas was investigated. The short-slot antenna operated within the frequency range of 900–1600 GHz, achieving a peak output power of 3.5 µW at 1250 GHz. Conversely, the long-slot antenna integrated with a UTC-PD primarily operated in the range of 350–850 GHz, reaching a maximum output power of 28 µW. Reference [12] explored an integration between a GaAs-AlGaAs-based UTC-PD and a sub-removed monopole antenna, demonstrating a peak output power of 20 mW within the frequency range of 100–250 GHz, with a 3 dB bandwidth exceeding 250 GHz. Another study described in the literature [13] involved integrating a UTC-PD with a log-periodic toothed antenna that achieved an output power of 2.3 µW at 1.04 THz and operated up to frequencies as high as 1.5 THz. Reference [14] described the integration of a UTC-PD with a bow-tie antenna, enabling operation up to frequencies as high as 2.5 THz; however, it exhibited a lower overall output power. A study [15] employed a GSG-configuration UTC-PD converted and integrated with planar antennas and achieved a low output power (−6 dBm) within the frequency range from 71 to 86 GHz. The studies referenced in [16,17], respectively, integrated TEM-mode horn antennas and dual-ridged horn antennas with UTC-PDs; however, narrow operating bandwidths were observed for these configurations. The work presented in the literature [18] integrated a UTC-PD with resonant antennas, resulting in power outputs ranging from 0.7 to 1.6 THz. These collective research findings provide valuable insights into integrating UTC-PDs with various linearly polarized antennas. However, there is a significant gap in the research regarding the integration with circularly polarized broadband antennas, which has significant implications for scenarios where the polarization direction is uncertain or changes over time.

This paper proposes a design for a circularly polarized UWB antenna that can be integrated with a UTC-PD. For the convenience of integrating the antenna with the UTC-PD later, the antenna design directly adopts the same form as the chip electrodes. The antenna achieves an impedance bandwidth of 100–1500 GHz (175%) and an axial ratio bandwidth of 150–720 GHz (131%). If integrated with a UTC-PD, the antenna can fully exploit the UWB advantages of the UTC-PD, greatly expanding the application range of the entire module.

## 2. Theory

The UTC-PD serves as a crucial source of THz radiation, and thus the output power of UTC-PD is highly valued. A theoretical analysis and curve fitting of the measured data [13] are employed to establish a model for the output power of the UTC-PD chip. The model includes the loss constant A and the bandwidth factor B.

(1) $$P_{\mathrm{RF}}=A\cdot B=\frac{A}{\left(1+(\frac{f}{BW_{CR}}{)}^{2})\cdot (1+(\frac{f}{BW_{TR}}{)}^{2}\right)}$$

The model incorporates several key parameters, including the operating frequency *f*, the CR-time-constant-limited bandwidth (*BW_CR_* = 1/2π*CR*), and the carrier transit-time-limited bandwidth (*BW_TR_*). By incorporating these parameters, a more detailed formula for the UTC-PD chip’s output power is derived, as shown in Equation (2).

(2) $$P_{\mathrm{RF}}=\frac{1}{2}R_{L}{\left(I_{0}^{id}\right)}^{2}\eta_{ext}^{2}\frac{1}{\left(1+{\left(\frac{f}{BW_{CR}}\right)}^{2})\cdot (1+{\left(\frac{f}{BW_{TR}}\right)}^{2}\right)}$$

where $R_{L}$ is the radiated resistor of UTC-PD integrated with antenna. $I_{0}^{id}$ is the current. $\eta_{ext}^{}$ is the corresponding external quantum efficiency. A more intuitive formula can be derived by further expanding the equation.

(3) $$\eta_{ext}=\frac{I_{0}^{id}/q}{P_{OP}/hf}=\frac{I_{0}^{id}}{P_{OP}}\cdot \frac{hf}{q}=r\cdot \frac{hf}{q}=rf\cdot \frac{6.63\cdot 10^{-34}}{1.6\cdot 10^{-19}}\approx rf\cdot 4.14\cdot 10^{-15}$$

(4) $$P_{RF}=\frac{1}{2}\cdot {\left(4.14\cdot 10^{-15}\right)}^{2}\cdot R_{L}\cdot P_{OP}^{2}\cdot r^{4}\cdot \frac{f^{2}}{\left(1+\frac{f^{2}}{BW_{CR}^{2}})\cdot (1+\frac{f^{2}}{BW_{TR}^{2}}\right)}$$

(5) $$BW_{CR}=\frac{1}{2\pi RC}=\frac{w_{c}}{2\pi R\varepsilon_{0}\varepsilon_{r}S}$$

(6) $$BW_{TR}=\frac{1}{2\pi (t_{drift}+t_{diff})}=\frac{1}{2\pi (\frac{w_{a}^{2}}{2D_{e}}+\frac{w_{a}}{v_{th}}+\frac{w_{c}}{v_{es}})}$$

where *S* is the area of the detector’s active layer, *w_a_* is the absorbing layer thickness, *w_c_* is the eigen zone thickness, *De* is the diffusion coefficient of electrons in the absorbing layer. *v_th_* is the hot-electron emission velocity at the edge of the absorption layer. And *v_es_* is the electron overshoot velocity in the collecting layer. It should be noted that all bandwidths mentioned in this paper refer to the edge-coupled UTC-PD. As indicated by Equation (4), the output power of the UTC-PD module is not only determined by the chip characteristics but also by the performance of the integrated antenna. Therefore, continuous optimization of the antenna performance is necessary to achieve optimal performance of the entire chip module in the future.

## 3. Design and Validation of a UWB Circularly Polarized Antenna for UTC-PD

**Figure 1:**
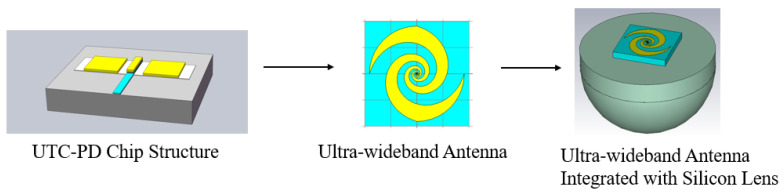
The design procedure of UWB circularly polarized UTC-PD module.

The integration process of the UWB circularly polarized antenna with the UTC-PD is depicted in Figure 1. After chip fabrication, the chip electrodes are directly integrated with the UWB circularly polarized antenna. The antenna structure is optimized to achieve better performance. Subsequently, this optimized structure is further integrated with the silicon lens to obtain the entire UWB circularly polarized UTC-PD module. The main focus of this paper remains on the design of the UWB circularly polarized antenna, and Figure 1 serves to illustrate the entire integration process.

### 3.1. UWB Circularly Polarized Spiral Antenna

**Figure 2:**
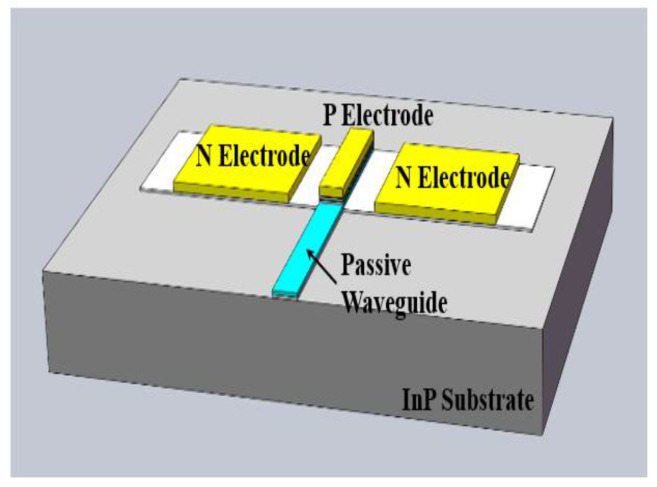
UTC-PD chip model.

Figure 2 shows the model of the UTC-PD chip, which includes the P electrode, N electrodes, passive waveguide, and InP substrate. The N-P-N electrode and passive waveguide are located on the InP substrate, and the planar electrode of the N-P-N facilitates subsequent integration, as discussed later. Table 1 lists six types of electrodes: The first type features a P electrode area of 3 × 15 µm^2^, an N electrode area of 15 × 15 µm^2^, and spacings between the P electrode and N electrodes of 4 µm, 5 µm, and 6 µm. The second type has a P electrode area of 3 × 25 µm^2^ and an N electrode area of 15 × 25 µm^2^ with the same spacings between the P electrode and N electrodes. Unlike the plane-incident UTC-PD, the passive waveguide of this edge-coupled UTC-PD directly points at the P electrode. As the input optical wave enters, it can be gradually absorbed, even with a thin absorbed layer, thereby achieving a high responsivity and wide response bandwidth simultaneously.

**Figure 3:**
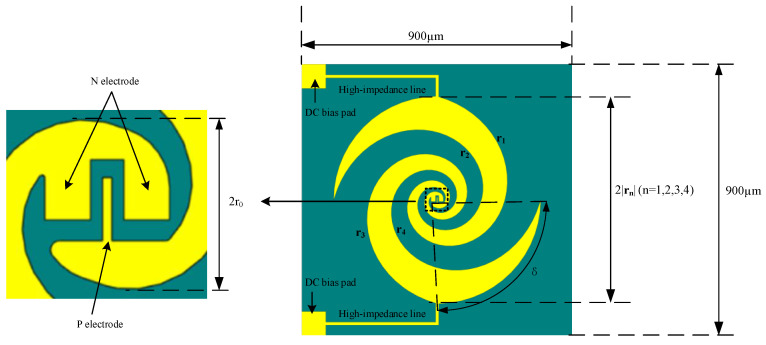
UWB cipcularly polarized spiral antenna.

The design of the spiral antenna combined with the UTC-PD chip electrode configuration is illustrated in Figure 3 (note that the UTC-PD chip has six different electrode sizes, with type A being exemplified here). The spiral antenna consists of four parts: the internal electrode shape, the DC bias pads, and two equiangular spiral arms. For the two arms, the corresponding definition expressions for their four sides are given by the following equations:

(7) $$r_{1}=r_{0}e^{a\phi }$$

(8) $$r_{2}=r_{0}e^{a\left(\phi -\delta \right)}$$

(9) $$r_{3}=r_{0}e^{a\left(\phi -\pi \right)}$$

(10) $$r_{4}=r_{0}e^{a\left(\phi -\delta -\pi \right)}$$

where r_0_ denotes the initial radius of the exponential curve, which is 30 µm; *a* represents the growth rate with a value of 0.27; and *ϕ* corresponds to the stop angle at which the curve ends, equaling 0.35π. To achieve broadband circular polarization, each arm of the spiral antenna is composed of two equiangular helices with a starting angle difference of *δ*, where *δ* is set to 90°, resulting in equal gap widths between adjacent arms and the spiral arm. The second arm is obtained by rotating the first arm by 180°, creating a highly symmetrical and self-complementary structure that facilitates easy attainment of broadband circular polarization.

The spiral antenna is designed to possess the same structure as the UTC-PD chip electrodes, enabling direct integration with the chip and eliminating the need for additional structures. Moreover, the antenna metal material consists of a 20 nm Ti layer and a 420 nm Au layer (notably, Ti is employed here to enhance the adhesion between Au and substrate), while employing a 100 µm InP substrate (ε = 12.56, tanD = 3 × 10^−4^).

**Figure 4:**
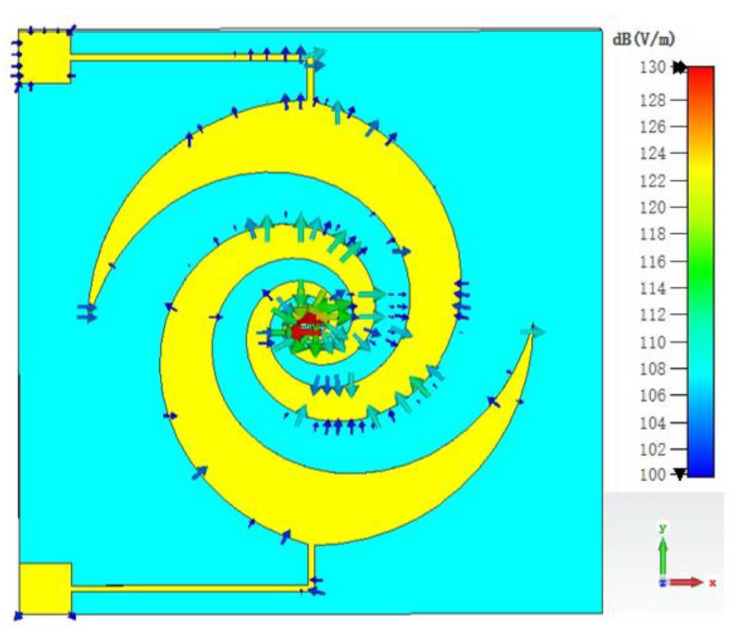
Electric field of antenna with thin high-impedance line (at 100 GHz).

**Figure 5:**
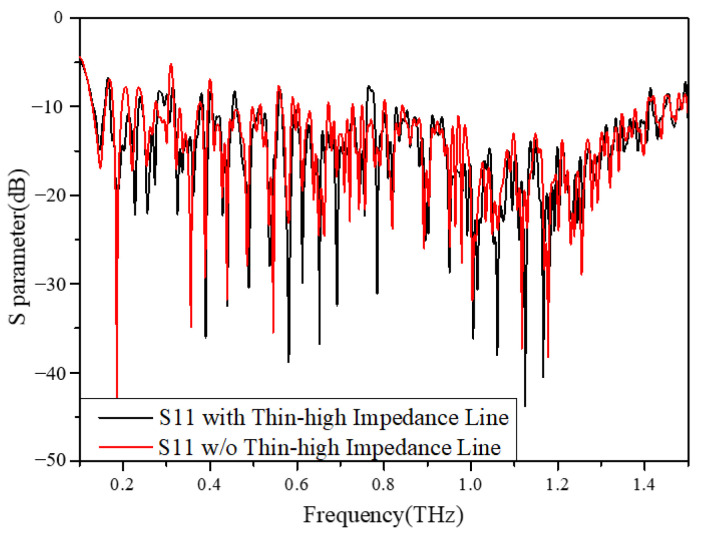
*S*_11_ of antenna with and without thin high-impedance line.

To provide DC bias, a thin, high-impedance line is connected to the outer arms of the spiral structure. This line has minimal impact on the overall antenna performance due to its narrow width, resulting in a low current flow and its positioning outside the effective radiation region. Since lower-frequency bands have larger radiation regions compared to higher frequencies, Figure 4 presents a simulated electric field distribution at 100 GHz with the presence of a high-impedance line using electromagnetic simulation software CST Studio Suite 2022. The results indicate that at the start of the thin high-impedance line, the electric field amplitude is decreased to 100 dB(V/m), which is 30 dB lower than that observed at the antenna’s starting body, thus confirming once again that the high-impedance line remains negligibly influential as it is located outside the effective radiation region. Conversely, when applying a bias voltage of −1 V or lower to this antenna, the DC voltage can reach the chip part directly. Furthermore, Figure 5 shows that impedance matching remains unaffected.

### 3.2. Optimization

The UTC-PD chip electrodes are in the N-P-N configuration, and the spiral antenna employs the same structure. While this monolithic integration method is convenient, it also introduces a new challenge: the internal symmetry of the spiral antenna is disrupted. As a result, impedance matching at the corresponding high frequencies deteriorates, with larger electrodes exacerbating this effect.

**Figure 6:**
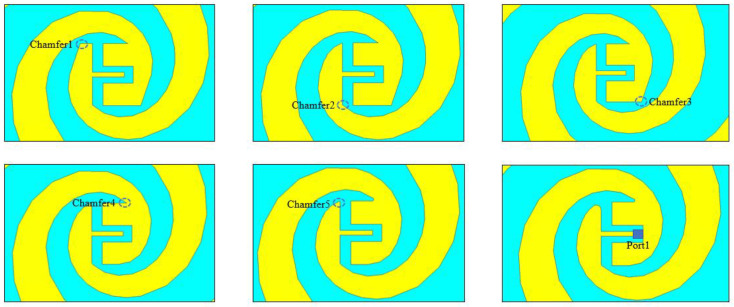
Optimization process of electrodes (type A).

**Figure 7:**
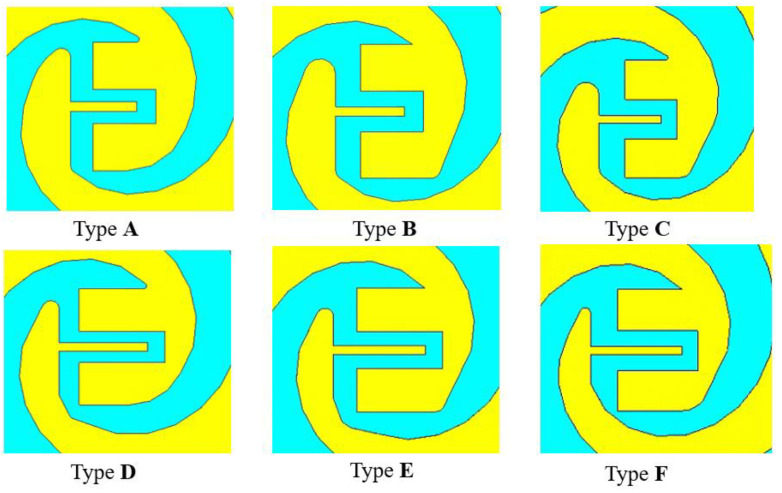
The final form of the six electrodes.

**Figure 8:**
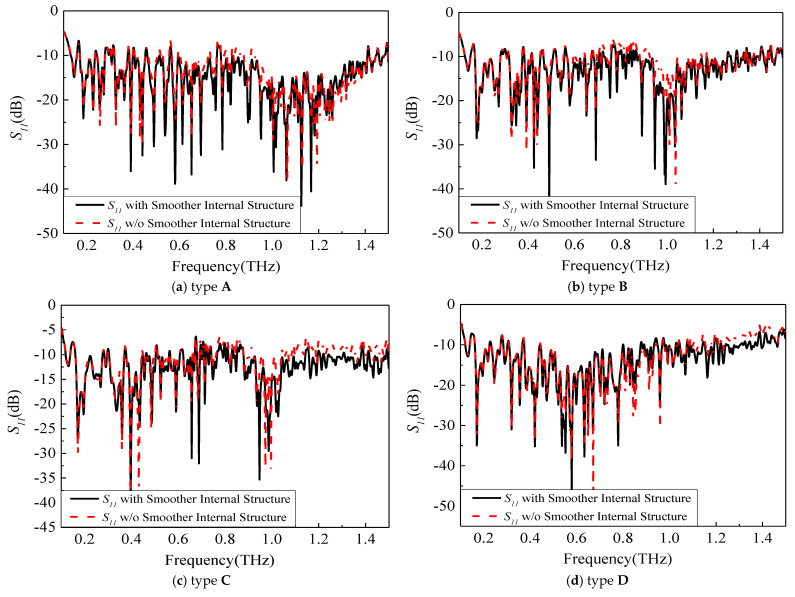

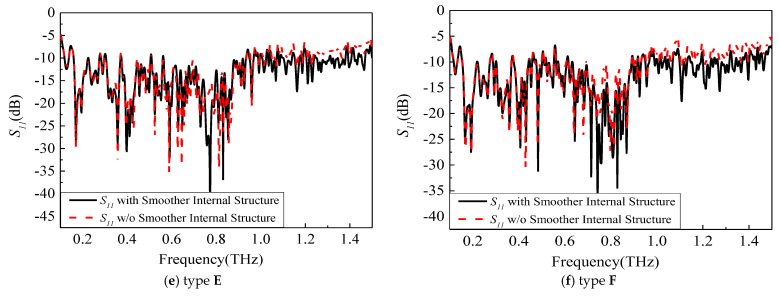
Comparison of *S*_11_ before and after electrode optimization.

To improve the internal symmetry broken by the chip electrodes’ form, it is possible to refine the internal structure of the antenna into a smoother structure. Figure 6, which uses type A as an example, shows how step-by-step chamfering can result in a more streamlined internal electrode structure and showcases the excitation port in this simulation. Furthermore, Figure 7 displays the final optimized configurations for all electrode types, which were optimized in a similar way to type A. The comparative results before and after smoothing are presented in Figure 8, revealing a notable enhancement primarily observed in the S parameter from 0.8 THz to 1.5 THz.

### 3.3. UWB Circularly Polarized Antenna Integrated with Silicon Lens

**Figure 9:**
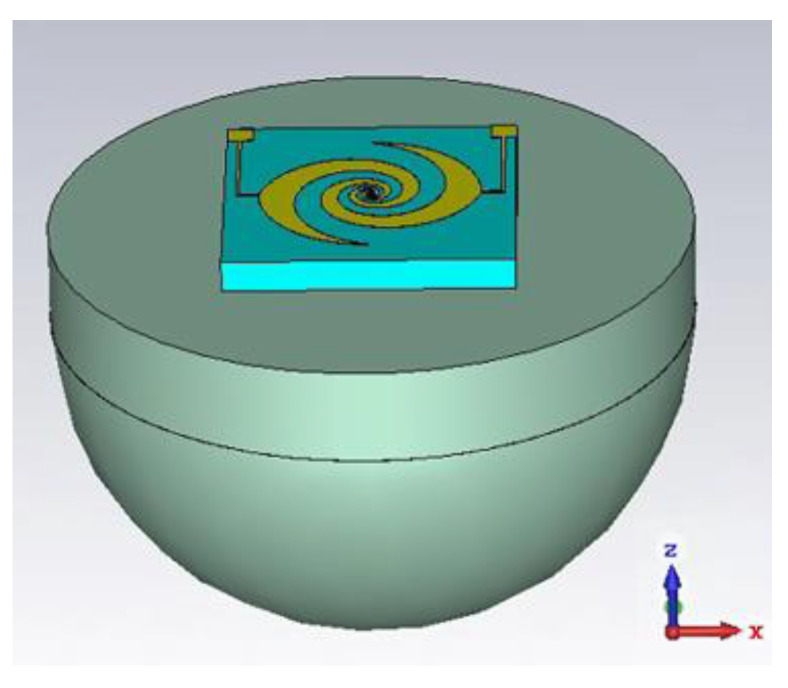
UWB circularly polarized antenna integrated with silicon lens.

**Figure 10:**
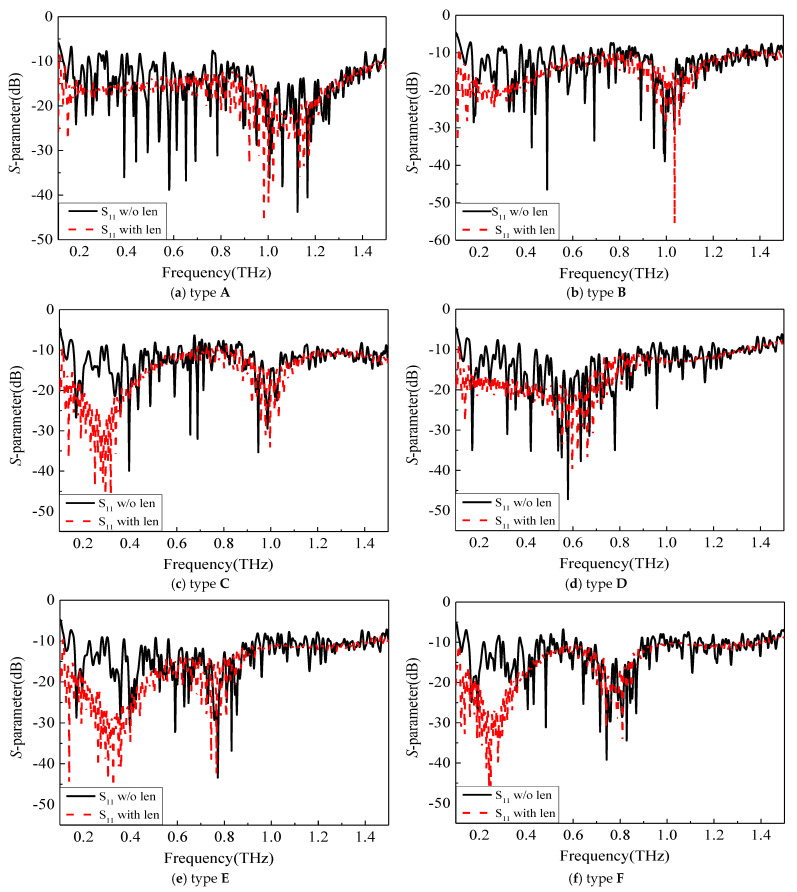
Comparison of *S*_11_ before and after integrated with silicon lens.

**Figure 11:**
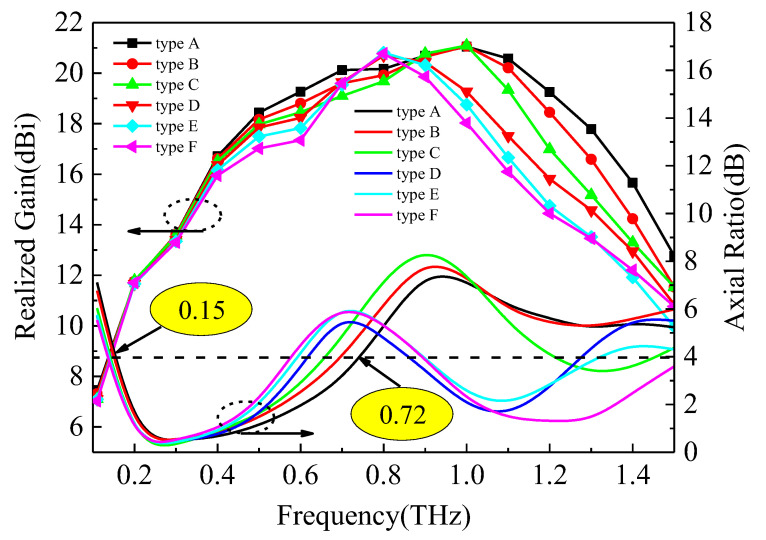
The realized gain and axial ratio of UWB antenna module.

**Figure 12:**
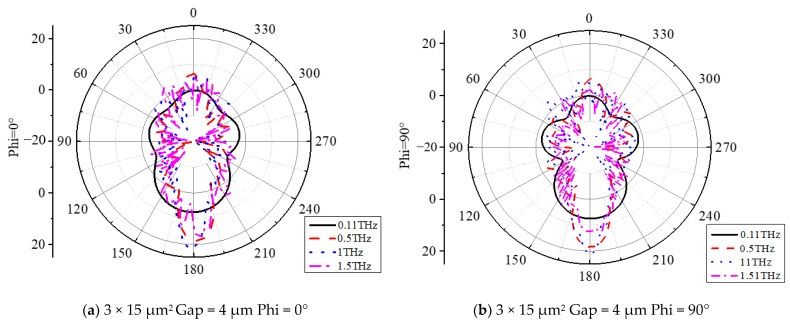
The antenna pattern of Phi = 0° and Phi = 90° at various frequencies.

To further enhance the performance of the antenna, a hyper-hemispherical silicon lens (with a diameter of 1 mm, bullet lens) was integrated with the UWB antenna, as shown in Figure 9. This dielectric lens increases the radiation directivity of the antenna and effectively suppresses surface waves. The impedance matching results before and after integration with the silicon lens are compared in Figure 10, demonstrating a significant improvement in overall module impedance matching. The reflection coefficient remains below −10 dB within the frequency range of 0.1–1.5 THz. The corresponding realized gain and axial ratio are presented in Figure 11. Different types of UWB circularly polarized antenna modules correspond to distinct internal electrode shapes. The low-frequency part corresponds to the external spiral structure, while the high-frequency part corresponds to the internal spiral structure. Therefore, the gain results of different types of low-frequency parts are not significantly different, but in the high-frequency part, the smaller the electrode shape, the higher the gain. These diverse UWB circularly polarized antenna modules achieved better impedance matching in the 0.8–1 THz range, resulting in an increase in gain followed by a decrease. Taking type A as an example, the maximum gain of 21.05 dBi was achieved at 1 THz. The 3 dB fractional bandwidth can reach 131% (0.15–0.72 THz). As shown in Figure 12, there are antenna patterns of Phi = 0° and Phi = 90° at various frequencies. The highest gain is at theta = 180°, and it can be observed that the lens has an obvious beam focusing effect. The silicon lens not only serves as a beam focusing function here but also acts as a dielectric resonator to improve the overall integrated radiation characteristics.

### 3.4. Experimental Results

**Figure 13:**
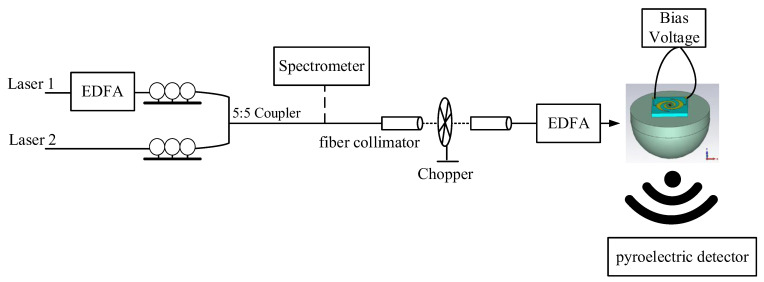
Measurement system of UWB antenna integrated with UTC-PD.

The measurement system of the UWB antenna integrated with the UTC-PD is illustrated in Figure 13. Two tunable lasers emit optical light with a specific wavelength difference and their polarization direction is adjusted using polarization controllers. The two optical lights are combined using a 5:5 coupler, and the correct combination wave is verified using a spectrometer. Subsequently, the wave undergoes chop modulation at a frequency of 15 Hz through an optical fiber collimator. It is then amplified by an Er-doped fiber amplifier (EDFA) and coupled into the UTC-PD via a taper fiber. The UTC-PD converts the optical signal into an electrical signal, ultimately detecting the power of THz frequency signals using a pyroelectric detector.

**Figure 14:**
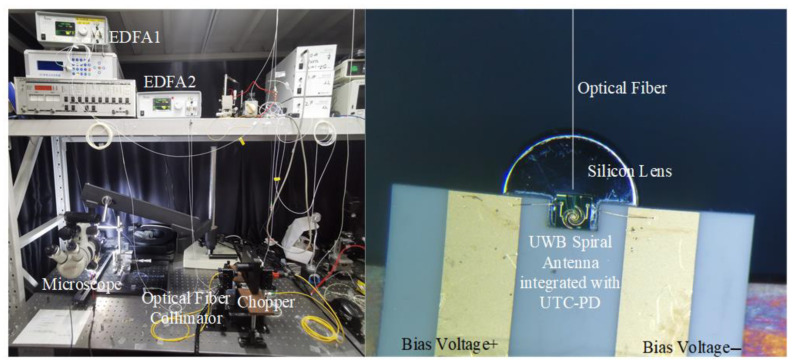
Experimental setup for THz power of UWB spiral antenna integrated with UTC-PD.

**Figure 15:**
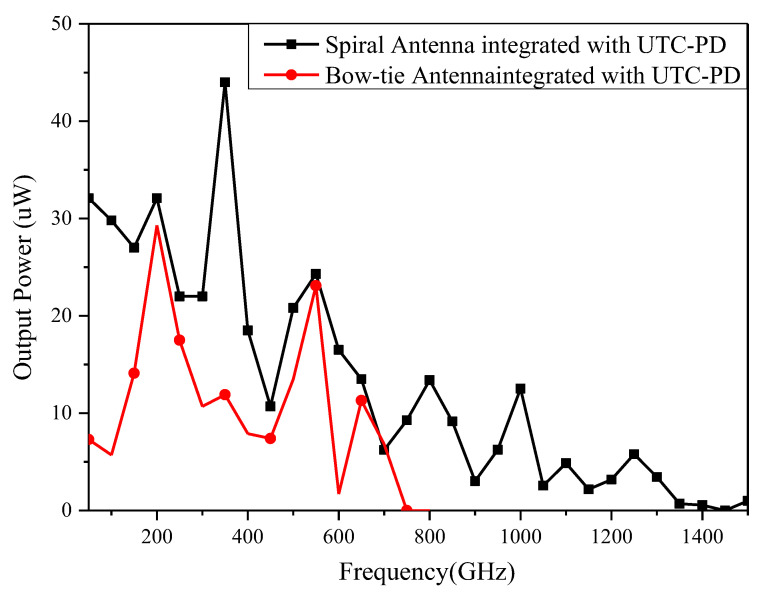
Measured THz power of UWB spiral antenna and bow-tie antenna integrated with UTC-PD as a function of frequency.

Figure 14 shows the experimental setup for measuring the THz power of the UWB spiral antenna integrated with the UTC-PD as a function of frequency. The corresponding measured results are presented in Figure 15. Both the bow-tie antenna and spiral antenna are integrated with the same fabrication process UTC-PD chip; however, the spiral antenna exhibits twice the bandwidth compared to the bow-tie antenna. This observation further confirms the superior UWB performance of the designed spiral antenna.

### 3.5. Comparisons

Table 2 summarizes the comparison of various antennas integrated with the UTC-PD. It is evident that this work has a broader operating frequency range and the added benefit of circular polarization, making it suitable for a wider range of applications.

## 4. Conclusions

This paper proposes a UWB circularly polarized antenna module for UTC-PD integration. The UWB circularly polarized antenna module can be directly integrated with the UTC-PD and has superior overall performance. By employing a modified planar spiral antenna, a 175% impedance bandwidth and 131% axial ratio bandwidth are achieved. The maximum gain of 21.05 dBi is obtained at 1 THz. The proposed module has the characteristics of a UWB, circular polarization, and easy application to THz applications.

## Figures and Tables

**Table 1:** Six types of electrodes.

	I			II		
	A	B	C	D	E	F
P Electrode	3 × 15 µm^2^	3 × 15 µm^2^	3 × 15 µm^2^	3 × 25 µm^2^	3 × 25 µm^2^	3 × 25 µm^2^
N Electrode	15 × 15 µm^2^	15 × 15 µm^2^	15 × 15 µm^2^	15 × 25 µm^2^	15 × 25 µm^2^	15 × 25 µm^2^
Gap_PN_	4 µm	5 µm	6 µm	4 µm	5 µm	6 µm

**Table 2:** Comparisons between different antennas integrated with UTC-PD.

Ref.	Antenna Type	Frequency (GHz)	Polarization
[11]	Short-slot antenna	900–1600 (56%)	Linearly
	Long-slot antenna	350–850 (83%)	
[14]	Bowtie antenna	300–2500 (157%)	Linearly
[13]	Log-periodic antenna	300–1500 (133%)	Linearly
This work	Planar spiral antenna	100–1500 (175%)	150–720 (131%) Circularly

## Data Availability

Data are contained within the article.
